# Association of Herd Size with Stillbirth and Dystocia Rates in Japanese Black Cattle

**DOI:** 10.3390/ani12151994

**Published:** 2022-08-06

**Authors:** Moe Misaka, Mizuho Uematsu, Go Kitahara, Takeshi Osawa, Yosuke Sasaki

**Affiliations:** 1 Course of Animal and Grassland Sciences, Graduate School of Agriculture, University of Miyazaki, Miyazaki 889-2192, Japan; 2 Miyazaki Agricultural Mutual Aid Association, Miyazaki 880-0852, Japan; 3 Department of Veterinary Sciences, Faculty of Agriculture, University of Miyazaki, Miyazaki 889-2192, Japan; 4 Center for Animal Disease Control, University of Miyazaki, Miyazaki 889-2192, Japan; 5 Department of Agriculture, School of Agriculture, Meiji University, Kanagawa 214-8571, Japan

**Keywords:** dystocia, epidemiology, health status, herd size, Japanese Black, stillbirth

## Abstract

**Simple Summary:**

Stillbirth and dystocia have a substantial impact on productivity and animal welfare in the beef industry. We previously elucidated risk factors for stillbirth and dystocia in the Japanese Black, the most common breed of beef cattle in Japan and known as Wagyu. Large variations in calving and calving management among different herd sizes were reported for beef cattle worldwide, but no published literature reported on the association between herd size and the stillbirth and dystocia rates in Japanese Black cattle. Here, we examined the effect of herd size on stillbirth and dystocia rates in Japanese Black cows. We found that the stillbirth rate was not associated with herd size, and the effect of herd size on stillbirth was independent of season, parity, and stage. In contrast, large farms had a lower dystocia rate than small and medium-sized farms, and higher dystocia on small farms compared to medium and large farms was found only in pregnancy of normal duration. This knowledge could contribute to designing and implementing an effective calving management system and finally decrease the burden on labor and clinical veterinarians.

**Abstract:**

The objective of this study was to investigate the effect of herd size on stillbirth and dystocia rates; the relationships between herd size, calving season, parity, and gestation length in Japanese Black cattle were also explored. Data were collected for 41,184 calvings from 15,512 animals on 905 farms between 2006 and 2010. In this study, herds were classified into three groups based on size: small (1–10 cows), medium (11–50 cows), and large (≥51 cows). Herd size had an effect on the dystocia rate (*p* < 0.05) but not the stillbirth rate. Additionally, interactions between herd size and gestation length were associated with the dystocia rate (*p* < 0.05), and the dystocia rate was the highest in small herds, followed by medium and large herds for cows with a gestation length of 281–300 days, which is considered a pregnancy of normal duration. In summary, in Japanese Black cattle, there were different effects of herd size on the stillbirth rate and dystocia rates, as herd size was associated with the dystocia rate but not with the stillbirth rate.

## 1. Introduction

Stillbirth and dystocia have a substantial impact on productivity and animal welfare in the beef industry and can result in direct economic losses due to both calf and dam death and premature culling, as well as indirect economic costs due to additional veterinary services, labor, and treatment [1]. In Japan, the most common breed of beef cattle is the Japanese Black, also known as Wagyu cattle. They have a superior ability over other breeds to produce marbled beef [2], and the value of a Japanese Black calf is approximately four times that of a Holstein–Friesian calf [3]. Our previous study reported that the incidences of stillbirth and dystocia were 2.46% and 8.55%, respectively [4]. Additionally, risk factors for stillbirth and dystocia in this breed were winter calving, primiparity, and short and long gestation lengths [4]. Furthermore, our previous study elucidated that Japanese Black cows with stillbirth and dystocia had inferior subsequent reproductive performance, such as a lower conception rate and higher occurrence of stillbirth at the subsequent calving than cows with normal calving [5].

The incidences of stillbirth and dystocia would be associated with management procedures. In the United States [6] and Japan [7], large variations in calving and calving management among different herd sizes were reported for beef cattle. In particular, Japanese Black herd sizes and the number of cows per farm have been increasing since 2005, when the mean number of cows per farm was 16, until in 2020, where it reached 40 [8,9,10,11] and proper management procedures in large herds should be constructed. The size of the herd influences the calving management procedures required and the stillbirth and dystocia rates. Associations between herd size and reproductive performance in Japanese Black cattle were investigated [12], but there is no published literature on the association between herd size and the stillbirth and dystocia rates in Japanese Black cattle. This knowledge could contribute to designing and implementing an effective calving management system and finally decrease the burden on labor and clinical veterinarians. Our objective was, therefore, to investigate the effect of herd size on stillbirth and dystocia rates and the interaction between herd size, calving season, parity, and gestation length in Japanese Black cattle.

## 2. Materials and Methods

Data used in this study were collected from farms in suburban areas of the city of Miyazaki in Miyazaki prefecture, Japan. Miyazaki prefecture is located on the southeastern coast of Kyushu, Japan; it is a major cow-calf producing region and has the second largest cattle population in the country. The data analyzed were a subset of a larger dataset from a previous study [4]. Records of 41,184 calvings that included both the calving and subsequent service events from 15,512 animals on 905 farms between April 2006 and March 2010 were used for the analysis. The mean number of adult cows per farm was 18 (range, 1–454). All dams had a 10-digit unique identification number. Dam number, the birth date of the dam, date of AI and calving, parity, gestation length, presence or absence of stillbirth or dystocia, and causes of dystocia were obtained from the database managed by the Miyazaki Prefecture Livestock Association. All animals were housed individually in a tie-stall barn where there was little difference in the inside vs. outside air temperature. Rice straw, Italian ryegrass, or oat straw was fed individually to cows twice daily. All animals were bred by artificial insemination (AI).

In this study, herd size was classified into three groups based on size: small (1–10 cows; *n* = 6770), medium (11–50; *n* = 19,210), and large (≥51; *n* = 15,204). Calving characteristics were classified into three groups: normal, stillbirth, or dystocia. Stillbirth was defined as a dead fetus at calving >240 days after AI [13], and dystocia was defined as a case in which the cow did not make progress more than 30 min after the rupture of the amniotic membrane and calving that required veterinary assistance. All diagnoses were made by 1 of 30 veterinarians because all obstetric interventions, however minor, were conducted by a veterinarian and, thus, included in the study. The calving month was categorized according to the season: winter (December to February), spring (March to May), summer (June to August), and autumn (September to November). A parity higher than 11 was combined with a parity of 10. The gestation length was divided into five groups: (1) ≤270 days; (2) 271–280 days; (3) 281–290 days; (4) 291–300 days; and (5) ≥301 days.

Data were analyzed using SAS software (v. 9.4) (SAS Institute Inc., Cary, NC, USA). A multilevel mixed-effects logistic regression model using the GLIMMIX procedure with contrasts was used to compare the stillbirth rate and dystocia rate between herds of different sizes and other parameters. The dependent variable was the probability of a stillbirth occurrence, investigated by whether or not a cow had a stillbirth (1 or 0), or the probability of dystocia, investigated by whether or not a cow had dystocia (1 or 0). The independent variables were herd size (small, medium, and large), calving season (winter, spring, summer, and autumn), parity (1, 2, 3, 4, 5, 6, 7, 8, 9, and ≥10), and gestation length (≤270, 271–280, 281–290, 291–300, and ≥301 days). All possible two-way interactions between herd size and other independent variables were included in all the models, but insignificant interactions (*p* ≥ 0.05) were removed from the final models. Farm and calving years were included as random effects in all models. The strength of association between the variables and the response was quantified by computing the odds ratios and their corresponding 95% confidence intervals (CI). Regarding herd size, either medium-sized or large farms were set as a reference. Regarding calving season and gestation length, summer and 291–300 days were set as references, respectively, based on the previous study (Uematsu et al., 2013). Regarding parity, parity 1, showing the highest stillbirth and dystocia rates, was set as a reference.

## 3. Results

The average stillbirth and dystocia rates for the 41,184 calvings were 2.2% and 8.5%, respectively. Average parity and gestation length were 4.9 days and 289.7 days, respectively. The average stillbirth rate in the small, medium, and large farms were 2.2%, 2.1%, and 2.2%, respectively. Stillbirth rate was associated with calving season, parity, and gestation length but not with herd size (*p* < 0.05; Table 1). Stillbirth rates in winter and spring were higher than in summer (odds ratio [95% CI]: 1.90 [1.55–2.33] and 1.49 [1.21–1.83], respectively). Cows in parity 1 had a higher stillbirth rate than those in parity 5 (1.47 [1.11–1.97]). Compared to cows that calved between 281 and 290 days of pregnancy, stillbirth rates were higher in cows that had been pregnant for ≥301 days (4.37 [2.89–6.61]), for ≤270 days (79.96 [62.83–101.76]), and for the cows that had been pregnant between 271 and 280 days (6.45 [5.16–8.05]). No interactions were observed between herd size and calving season, parity, or gestation length that affected the stillbirth rate.

The dystocia rate was associated with herd size, calving season, parity, and gestation length (*p* < 0.05). The dystocia rate on large farms (mean: 3.6%) was lower than that on medium-sized farms (10.4%) and small farms (14.0%), but there was no significant difference between rates on small farms and medium-sized farms (Table 1). Dystocia rates in winter and spring were higher than in summer seasons (1.28 [1.15–1.43] and 1.36 [1.23–1.51], respectively). Cows in parity 1 had a higher stillbirth rate than those in parity 5 (2.12 [1.78–2.53]). Compared to cows that calved between 281 and 290 days of pregnancy, stillbirth rates were higher in cows that had been pregnant for ≥301 days (1.59 [1.12–2.25]), for ≤270 days (2.89 [2.09–4.00]), and for cows that had been pregnant between 271 and 280 days (2.16 [1.80–2.60]).

Additionally, interactions between herd size and parity or gestation length were associated with dystocia rate (*p* < 0.05). The interaction between herd size and parity had an effect on the dystocia rate (*p* < 0.05), but the effect of herd size in each parity and the effect of parity in each herd size were similar to the main effects. Table 2 shows the results of the comparison of the dystocia rate between herd sizes in each gestation length and the comparison of dystocia rate between each gestation length in each herd size group. Regarding the interaction between herd size and gestation length, cows on large farms had a lower dystocia rate than those on medium farms for all gestation lengths. However, small farms had a similar dystocia rate to medium farms for gestation lengths <280 days and >301 days, whereas there was a higher dystocia rate for a gestation length of 281–300 days.

## 4. Discussion

To the best of our knowledge, this study is the first to report on the association between herd size and stillbirth and dystocia rates in Japanese Black cattle. In this study, the effect of calving season, parity, and gestation length on the stillbirth rate and dystocia rate was consistent with a previous study [4], but the effect of herd size was not: herd size was associated with the dystocia rate but not with the stillbirth rate. This is because stillbirth is mainly due to biological factors, whereas dystocia is mainly the consequence of anthropogenic factors such as calving management and the frequency of calving assistance and observation by a veterinarian, which reportedly differ among herd sizes of Japanese Black cattle [7]. Therefore, the difference in the dystocia rate among different herd sizes would be affected by calving management. It is important for producers and researchers to pay attention when using stillbirth and dystocia rates because these indicators have different meanings.

The dystocia rate of Japanese Black cattle was the highest in small herds, followed by medium-sized and large herds for cows with a gestation length of 281–300 days, which is considered a pregnancy of normal duration. Our results indicated that the smaller the herd size, the more often the farmers required veterinarians to visit their farms to assist in calving. This difference may be due to farmers being conscious of the calving risks and the frequency of calving assistances and observations. Our previous study reported that a higher proportion of large farms had remote censors, such as vaginal temperature sensors and automated monitoring devices, to predict calving times than small farms [7]. The application of ICT devices can decrease the accident rate at calving from 2.2% to 0.3% [14], and calving devices are effective for predicting the calving time and reducing the risk of fetal death [15]. In postpartum dairy cattle, small and medium-sized herds required veterinarians to visit the herd “only if needed” compared with large herds [16]. A higher frequency of veterinarian visits and medical prescriptions results in an increase in costs. Thus, we recommend that producers check carefully to see whether dystocia has occurred before requesting veterinary visits. It is important to re-evaluate the criteria for the requirement of veterinary assistance.

In the present study, herd size was not associated with the stillbirth rate, and interactions between herd size and calving season, parity, and gestation length were not associated with the stillbirth rate. These results indicated that the effect of herd size on the stillbirth rate was at least partially independent of anthropogenic factors and calving management. Therefore, decreasing the stillbirth rate by improving standard operational procedures and calving management strategies is difficult. To decrease the stillbirth rate, one option is to take care of cows calving in winter. This period reportedly carries a high risk of stillbirth because the birth weight of calves increases with a drop in temperature, resulting in an increase in calving difficulty [17]. A previous study [17] reported that as average winter temperatures decreased, subsequent calf birth weights increased, and calving difficulty increased. In addition, one of the factors associated with an increase in stillbirth rates in the winter would be both stillbirth and dystocia due to abnormal fetal positions [4]. An abnormal fetal position might be caused by fetal death in utero before the onset of parturition, and a greater number of fetuses might have died in the winter before calving. Further studies will be required to determine whether, in Japanese Black cattle, there is an association between calf birth weights and stillbirth/dystocia rates with temperature.

The present study had several limitations that should be noted when interpreting the results. First, a possible variable, such as nutritional condition, which we could not evaluate in this study, might have influenced the results. Second, we could not evaluate farmers’ attitudes to the calving conditions in each herd size. Further studies will be required to determine whether there is an association between different farmers’ attitudes or nutritional conditions among Japanese Black cattle herd size groups and the dystocia rate. Lastly, the data in this study are about a decade old. Although the environment and management have not dramatically changed since then, it may be necessary to be cautious about whether the results reflect the current situation.

## 5. Conclusions

In summary, herd size was not associated with the stillbirth rate, and there was no effect of interactions between herd size and calving season, parity, and gestation length on the stillbirth rate in this cohort of Japanese Black cattle. However, the dystocia rate was the highest in small herds, followed by medium-sized and large herds of cows with a pregnancy of a normal duration of 281–300 days.

## Figures and Tables

**Table 1:** Stillbirth and dystocia rate by herd size, season, parity, and gestation length.

		Stillbirth Rate, %		Dystocia Rate, %	
	Number of Animals	Mean	OR (95% CI) ^1^	Mean	OR (95% CI) ^1^
Herd size ^2^ (Medium-sized farm set as reference)					
Small	6770	2.2	NS ^3^	14.0	NS ^3^
Medium	19,210	2.1		10.4	Reference
Large	15,204	2.2		3.6	0.28 (0.20–0.40)
Herd size ^2^ (Large farm set as reference)					
Small	6770	2.2	NS ^3^	14.0	4.03 (2.73–5.95)
Medium	19,210	2.1		10.4	3.59 (2.52–5.11)
Large	15,204	2.2		3.6	Reference
Calving season ^4^					
Winter	9678	2.9	1.90 (1.55–2.33)	9.0	1.28 (1.15–1.43)
Spring	11,127	2.3	1.49 (1.21–1.83)	9.9	1.36 (1.23–1.51)
Summer	11,378	1.6	Reference	7.8	Reference
Autumn	9001	1.8	NS ^3^	7.0	NS ^3^
Parity					
1	5854	3.2	Reference	12.9	Reference
2	5304	2.0	0.65 (0.50–0.85)	7.6	0.52 (0.44–0.60)
3	4825	2.0	0.67 (0.51–0.88)	8.1	0.56 (0.48–0.65)
4	4511	2.1	0.72 (0.54–0.95)	7.2	0.45 (0.38–0.53)
5	4229	1.9	0.68 (0.51–0.91)	7.8	0.47 (0.40–0.56)
6	4091	1.3	0.48 (0.35–0.67)	7.8	0.50 (0.42–0.60)
7	3742	2.1	0.76 (0.57–1.01)	8.0	0.50 (0.42–0.59)
8	3201	1.9	0.63 (0.46–0.87)	7.2	0.45 (0.37–0.55)
9	2375	2.4	0.77 (0.55–1.07)	7.2	0.40 (0.31–0.50)
≥10	3052	2.3	0.66 (0.48–0.90)	8.6	0.56 (0.46–0.67)
Gestation length, days					
≤270	379	53.6	79.96 (62.83–101.76)	21.1	2.89 (2.09–4.00)
271–280	1447	8.6	6.45 (5.16–8.05)	14.4	2.16 (1.80–2.60)
281–290	19,883	1.5	Reference	8.6	Reference
291–300	19,023	1.3	NS ^3^	7.5	NS ^3^
≥301	452	6.0	4.37 (2.89–6.61)	11.1	1.59 (1.12–2.25)

^1^ OR (95% CI): odds ratio (95% confidence interval); ^2^ herd size: small (1–10 cows), medium-sized (11–50 cows), large (≥51 cows); ^3^ NS: not significant; ^4^ calving season: winter (December–February), spring (March–May), summer (June–August), and autumn (September–November).

**Table 2:** Comparison of dystocia rate between herd sizes in each gestation length and comparison of dystocia rate between each gestation length in herd size group.

Gestation Length, Days	Herd Size ^1^								
	Small			Medium			Large		
	N	Mean	OR (95% CI) ^2^	N	Mean	OR (95%CI) ^2^	N	Mean	OR (95% CI) ^2^
Comparison of dystocia rate between herd size in each gestation length (medium-sized farm set as reference)									
≤270	52	21.2	NS ^3^	185	25.9	Reference	142	14.8	0.40 (0.21–0.79)
271–280	196	25.0	NS ^3^	539	20.6		712	6.9	0.22 (0.13–0.35)
281–290	3157	14.5	1.43 (1.17–1.74)	9082	10.9		7644	3.5	0.24 (0.17–0.33)
291–300	3293	12.7	1.69 (1.38–2.08)	9218	8.8		6512	3.1	0.31 (0.23–0.44)
≥301	72	18.1	NS ^3^	186	14.5		194	5.2	0.26 (0.11–0.59)
Comparison of dystocia rate between herd size in each gestation length (large farm set as reference)									
≤270	52	21.2	NS ^3^	185	25.9	2.49 (1.27–4.87)	142	14.8	Reference
271–280	196	25.0	5.34 (3.08–9.28)	539	20.6	4.60 (2.84–7.43)	712	6.9	
281–290	3157	14.5	5.98 (4.32–8.27)	9082	10.9	4.19 (3.05–5.77)	7644	3.5	
291–300	3293	12.7	5.37 (3.87–7.47)	9218	8.8	3.18 (2.30–4.39)	6512	3.1	
≥301	72	18.1	4.77 (1.81–12.54)	186	14.5	3.90 (1.69–9.01)	194	5.2	
Comparison of dystocia rate between each gestation length in herd size group									
≤270	52	21.2	79.96 (62.83–101.76)	185	25.9	3.40 (2.35–4.91)	142	14.8	5.72 (3.43–9.54)
271–280	196	25.0	6.45 (5.16–8.05)	539	20.6	2.39 (1.88–3.03)	712	6.9	2.18 (1.56–3.03)
281–290	3157	14.5	Reference	9082	10.9	Reference	7644	3.5	Reference
291–300	3293	12.7	NS ^3^	9218	8.8	0.70 (0.63–0.79)	6512	3.1	NS ^3^
≥301	72	18.1	4.37 (2.89–6.61)	186	14.5	1.63 (1.05–2.54)	194	5.2	NS ^3^

^1^ Herd size: small (1–10 cows), medium-sized (11–50 cows), large (≥51 cows); ^2^ OR (95% CI): odds ratio (95% confidence interval); ^3^ NS: not significant.

## Data Availability

The data presented in this study are available upon request from the corresponding author. The data are not publicly available due to privacy.
